# ‘Digital Insight and Agency Scale’ (DIAS): A Novel Tool to Illuminate Young People's Agency in Mitigating the Negative Impact of Digital Activities on Their Mental Health

**DOI:** 10.1002/mpr.70053

**Published:** 2026-02-09

**Authors:** P. Tang, K. Kostyrka‐Allchorne, J. Bourgaize, A. Murray, M. Stoilova, M. E. Etherson, E. Azeri, I. Abbas, A. Bridgwood, C. Hollis, E. Townsend, S. Livingstone, E. J. S. Sonuga‐Barke, Digital Youth Research Programme

**Affiliations:** ^1^ Department of Psychology School of Biological and Behavioural Sciences Queen Mary University of London London UK; ^2^ Department of Child and Adolescent Psychiatry Institute of Psychiatry Psychology & Neuroscience King's College London London UK; ^3^ Clinical Outcome Solutions Folkestone UK; ^4^ School of Psychology University of Edinburgh Edinburgh UK; ^5^ Department of Media and Communications London School of Economics and Political Science London UK; ^6^ School of Health and Wellbeing University of Glasgow Glasgow UK; ^7^ East London NHS Foundation Trust London UK; ^8^ National Institute of Health Research (NIHR) MindTech MedTech Research Centre Institute of Mental Health School of Medicine University of Nottingham Nottingham UK; ^9^ School of Psychology University of Nottingham Nottingham UK

**Keywords:** adolescence, agency, anxiety, coping, depression, digital risk perception and management

## Abstract

**Objectives:**

Excessive time spent online is believed to negatively impact youth mental health; however, simplified screen‐time measures fail to consider young people's agency and digital activity management skills. We developed and validated a novel tool, the *Digital Insight and Agency Scale* (DIAS), to better understand different aspects of young people's online agency and explore their links to youth mental health.

**Methods:**

Participants (*n* = 383; age 16–25 years, mean = 19.0, SD = 1.7; 48.8% White, 30.2% South/East Asian, 8.6% Black) completed the DIAS questionnaire and standardised measures of anxiety, depression and wellbeing. The factor structure, reliability of the DIAS and associations with mental health were examined.

**Results:**

Participants reported specific negative impacts of digital engagement on their daily functioning in the previous 2 weeks, especially less sleep. Seventy‐eight per cent were worried about the negative impact of digital activity, and 82% engaged in one or more risk management actions, including *Enhancing Positive Engagement*, *Coping Actions*, and/or *Reducing Engagement*. Higher levels of mental health problems were associated with more worries and increased efforts to manage digital activity.

**Conclusions:**

Most young people displayed agency in managing their digital activity, suggesting that this could be leveraged in interventions, rather than focusing solely on reducing access and time spent online.

## 1 Introduction

Digital technology is becoming an irreplaceable aspect of young people's ‘always‐online’ lives (Szymkowiak et al. 2021), with 98% of 13–17‐year‐olds in the UK owning a mobile phone (Ofcom 2025). Across 19 European countries, 15–16‐year‐olds spend 3.8 h online each day (Smahel et al. 2020). In the US, over 80% of 12–17‐year‐olds spend two or more hours on their phones daily (Centers for Disease Control and Prevention 2022). While digital activity has clear benefits for young people, bringing opportunities for social connection, support, entertainment and personal development (Hollis et al. 2020), concerns are growing about the negative impact of excessive time online on daily functioning and mental health (Odgers and Jensen 2020).

Interventions to improve adolescent mental health often focus on reducing the time spent online or banning access entirely for under–16s (Barthorpe et al. 2020; Goodyear, Randhawa, et al. 2025b; Hale and Guan 2015; Malinauskas and Malinauskiene 2019). Such approaches, however, are unlikely to succeed (Livingstone et al. 2022; Goodyear, James, et al. 2025a) partly because the association between screen time and mental health is itself inconsistent and modest (Keles et al. 2020; Kerr et al. 2025; Kostyrka‐Allchorne et al. 2022; Odgers and Jensen 2020; Orben 2020). Indeed, adolescent depression and anxiety symptoms are better predicted by *specific* digital risk activities rather than by screen time in general (Sonuga‐Barke et al. 2024), likely because screen time may also be spent on activities that promote wellbeing (Winstone et al. 2021, 2021/09/24). Also emerging as important to wellbeing are the ability to distinguish between passive and active use (Niu et al. 2025), exposure to digital risks versus positive engagement (Kostyrka‐Allchorne et al. 2025), and the subjective reactions of adolescents to their digital engagement (Janssen et al. 2025), including their sense of control and self‐image (Liu et al. 2025; Moreno‐Padilla et al. 2025; Yang et al. 2025). Practically speaking, restrictive approaches are at odds with the reality of young people's digital lives, where digital technology is relied on for social, informational and educational activities (Ofcom 2025). For such reasons, restricting online engagement of vulnerable youth could even be counterproductive, harming mental health (Kostyrka‐Allchorne et al. 2022).

Recent theories propose a reciprocal and multifaceted relationship between technology use and mental health (Janssen et al. 2025; Sonuga‐Barke et al. 2024; Thabrew and Gega 2023), such that poor mental health can result in riskier modes of digital engagement, which in turn can worsen mental health. Kostyrka‐Allchorne et al. (2025) showed not only that exposure to risky digital activities was associated with depressive symptoms, but also that the results depended on the extent to which such risk exposure induced negative cognitive and emotional reactions. Recognising both the negative impacts of specific risk exposure (Livingstone and Smith 2014; Smahel et al. 2025) and individual variation in thoughts, feelings, and behaviours resulting from digital media use (Livingstone et al. 2025; Pouwels et al. 2021), we further propose a role for young people's capacity to mitigate risk (Sonuga‐Barke et al. 2024). The extent to which they spontaneously mitigate the negative impacts of digital activity on their mental health is hypothesised to depend on (i) recognising the negative impact and (ii) finding strategies to mitigate these impacts.

There is growing evidence that young people may be aware of the risks of digital activity, for example, they report concerns about the adverse impact on sleep (MacKenzie et al. 2022), academic performance (Mulisa and Getahun 2018), and family and social relationships (Allen et al. 2014). One recent review revealed young people's insights into how digital activities could undermine their mental health (Popat and Tarrant 2023). Related research has investigated adolescents' strategies for self‐regulating their digital activities and coping with online risks (Lorenzo et al. 2022; Vissenberg et al. 2022, 2022/04/01; Yu et al. 2019). Such evidence could warrant interventions that provide young people with the skills to improve digital resilience, defined by the UK Council for Internet Safety (2019, 4) as a ‘*dynamic personality asset that grows from digital activation, rather than through avoidance and safety behaviours*’. Promoting spontaneous coping and a sense of control, insight and agency are critical components of resilience in the face of stress and adversity (Hammond et al. 2024; Helsper et al. 2024; Livingstone et al. 2023; Pan et al. 2024; Rikala 2020; Rutter 2013).

However, before investing in such interventions, we need a better understanding of young people's strategies for managing their digital engagement and reducing the negative impacts. Currently, little is known about whether recognition of digital risks is associated with *spontaneous* attempts to change patterns of digital activity to reduce those risks. Contrasting with actions prompted by educators and caregivers, this spontaneity represents the ability of young people to recognise and reflect on a problem and subsequently take proactive actions. The few qualitative studies that have been conducted suggest that young people, once they have become aware of the risks of digital activity to their mental health, do try to take some action to reduce them, including seeking emotional support from others, altering their digital habits, or complying with parental/school rules on digital use as a protection (Livingstone et al. 2022; Schmidt et al. 2021; E. C. Weinstein et al. 2015; E. Weinstein 2018). However, having an awareness of the negative impacts of digital activity (e.g., on sleep) may be insufficient to motivate young people to take appropriate action (MacKenzie et al. 2022). Moreover, there are no existing validated measures of awareness of and actions to mitigate digital risks.

To address this gap, this paper reports on the development of a new questionnaire, *Digital Insight and Agency Scale* (DIAS), co‐produced with adolescents to assess their insight into, and attempts to manage, the impact of online experiences on daily functioning and mental health. We addressed six research questions:

1. What proportion of young people in the study perceive that their online experiences impacted their daily functioning and mental health? Are they concerned about this?
2. What proportion of young people in the study have attempted to modify/manage their online experiences to reduce the negative impact on their mental health?
3. Do the different risk management actions form distinct groupings? Were some types of actions more frequently employed than others?
4. Are young people who perceive a greater impact of online experiences on their mental health and daily functioning more likely to have attempted to modify/manage their online experiences?
5. Is engagement in risky digital activity related to concerns about the impact, and levels of engagement in risk management actions? Are these effects exacerbated in individuals with mental ill‐health (i.e., depressive and anxiety symptoms)?
6. Do these effects extend from mental ill‐health to wellbeing?

## 2 Methods

### 2.1 Participants

Two samples were recruited from UK secondary schools and universities. In the first sample, participants were aged 16–20 years; in the second sample, participants were aged 18–25 years. In total, 383 young people (mean age = 19.0 years, SD = 1.7) were recruited, of whom 75.5% were female, and 23.8% were male. The sample represented diverse ethnic backgrounds, with 48.8% White, 25.8% South Asian, 8.6% Black, 4.4% East Asian, and 10.5% having mixed or other ethnicities. Overall, 16.8% of the sample were eligible for free school meals at any point—an accepted UK indicator of socioeconomic disadvantage. This is below the national average of 24.6% in 2024 (Department for Education 2024). For a detailed description of the sample, please see Kostyrka‐Allchorne et al. (2025).

### 2.2 Measures

The *Digital Insight and Agency Scale* (DIAS) measures young people's awareness of and attempts to manage the impact of their online experiences on their daily functioning and mental health. The items were co‐developed with a panel of young people aged 12–17 (*n* = 14), drawing on the multidisciplinary expertise of digital media and youth mental health studies in the study team. A list of candidate items was drawn up by the team and then reviewed by the youth panel regarding whether the items were relevant and covered a comprehensive range of online experiences. The co‐design process was described in a separate publication (Kostyrka‐Allchorne et al. 2025).

The DIAS has two sections: (i) the perceived impact of online experiences on one's daily functioning and mental health over the preceding two weeks (9 items) and (ii) actions taken to manage those impacts in the same period (14 items). In section one, participants were asked ‘In the last 2 weeks, has your online experience affected your everyday life?’. Six items captured daily functioning impacts, including ‘I missed meals’, ‘I slept less’, and ‘I had difficulties at school’, etc. Answers were rated on a 5‐point scale (0 = never, 1 = just once or twice, 2 = a few times a week, 3 = most days, and 4 = at least every day). Three items related to participants' perceptions of how online experiences have affected their mental health, by asking ‘In the last 2 weeks, has being online affected you in any of the following ways?’, including ‘Being online positively affected my mental health’, ‘Being online negatively affected my mental health’, and ‘I worried how being online affected my mental health’, each answered on a 5‐point scale (0 = not at all, 1 = slightly, 2 = somewhat, 3 = quite a lot, and 4 = very much).

Section two measured whether respondents engaged in any actions because they were worried about how being online affected their mental health, by asking ‘In the past 2 weeks, did you do any of the following because you worried about how being online was affecting your mental health?’. It described specific actions that young people could have taken to mitigate the negative impact. Items included ‘Took a break from social media’, ‘Avoided upsetting content’, ‘Looked for more positive content’, and ‘Tried to engage with positive content’. Young people rated how often they had undertaken these actions on a 5‐point scale from 0 = never to 4 = at least every day, the same as the functional impact items.

#### 2.2.1 Risky Digital Activity

The *Digital Activities and Feelings Inventory* (DAFI; Kostyrka‐Allchorne et al. 2025) is a newly developed questionnaire designed to capture digital activities most relevant to young people. Section one of the DAFI contains 23 items which measure five distinct types of digital activities. Young people rated how often they experienced each item in the past 2 weeks on a 5‐point scale from 0 = never to 4 = at least every day. The DAFI has demonstrated good test‐retest reliability. The three subscales associated with mental health risk (risky content, risky interactions, and social comparison) were combined to form a single risk variable.

#### 2.2.2 Depression, Anxiety and Wellbeing

Measures of depression and anxiety symptoms were different for the two samples. The younger sample completed the Revised Child Anxiety and Depression Scale 25—Youth Version (RCADS‐25), a 25‐item scale with 10 items on depression and 15 items on anxiety. Participants rated how often each statement happened to them on a 4‐point scale from 0 = never to 3 = always. Total scores for depression and anxiety were calculated separately, with higher scores indicating more severe symptoms. The older sample completed the Depression, Anxiety, and Stress Scale (DASS‐21). The respective depression and anxiety subscales consist of seven items each. Participants rated them on a 4‐point scale from 0 = did not apply to me at all to 3 = applied to me very much or most of the time over the past week. Scores for individual items were summed separately for depression and anxiety, with higher scores indicating more severe symptoms.

Wellbeing was measured by the Warwick‐Edinburgh Mental Wellbeing Scale (WEMWBS) in both samples. The 14‐item WEMWBS measures positive feelings and psychological functioning and was answered on a 5‐point scale from 0 = none of the time to 5 = all of the time. A total score was calculated by summing the items, with higher scores indicating better mental wellbeing.

### 2.3 Procedure

All participants provided written consent before completing the study online via Qualtrics (https://www.qualtrics.com; the younger sample) or Gorilla (https://www.gorilla.sc; the older sample) between April 2023 and March 2024. Participants completed the survey in their own time and could skip questions they did not wish to answer. The younger sample completed the DIAS twice, approximately 1 week apart, to allow for test‐retest analysis. Depending on their preference, participants received a £10 shopping voucher or three course credits (the older sample only) as compensation for participating in the study. Ethical approval was received from the London School of Economics and Political Science for the younger sample (reference 18934) and Queen Mary University of London (reference PSY2023‐39A) for the older sample.

### 2.4 Analysis Strategy

To answer research question one, we calculated the proportion of participants who perceived an impact of their digital activity on daily functioning and mental health. For impacts on daily functioning, we contrasted those who reported an impact to happen (a) *never*, with (b) *just once or twice/a few times a week,* and (c) *most days/at least every day*. For mental health impacts and worry we contrasted those who reported (a) *no impact at all*, with those who reported (b) *slightly/somewhat impactful* and (c) having *quite a lot of impact/very impactful*.

To answer research question two, the proportion of participants who adopted various risk management actions was calculated by contrasting: (a) *never,* (b) *just once or twice/a few times a week* and (c) most *days/at least every day*.

To answer research question three, we performed exploratory factor analyses (EFA) using principal axis factoring with direct oblimin rotation (an oblique rotation that allows the factors to correlate) to examine the factor structure of the DIAS. The number of extracted factors was identified based on the Kaiser criterion (eigenvalue > 1), visual inspection of a scree plot, the proportion of variance explained by each factor, and the conceptual coherence of the factor solutions. Subscale scores were then calculated by averaging individual scores of the loaded items, where items that cross‐loaded (loadings >| 0.4|) or had small loadings (<|0.4|) were excluded. This was designed to achieve a measure that conforms to a simple structure to enhance its interpretability. Cronbach's alpha was calculated to examine the internal consistency of each factor. Individual differences in risk management according to biological sex or socioeconomic status were tested using independent sample *t*‐tests. Test‐retest reliability of the factors was calculated in the younger sample. A one‐way ANOVA was conducted to test the difference in means of the factors.

To answer research question four, we used Pearson's correlations to examine the relationships between the perceived impact of digital activity and risk management actions. We conducted an EFA of the functional impact items. An aggregated functional impact score was calculated by summing the scores of the items.

To answer research questions five and six, we first used Pearson's correlations to examine the relationships between perceived impact and risk management actions with depression and anxiety levels and risky digital exposure. Scores of RCADS‐25 and DASS‐21 were Z‐transformed before being used in the analyses. We then conducted multiple regressions to test the independent contribution of depression, anxiety, and wellbeing and risky digital activity to engaging in attempts to manage digital activities (main effects). We then added interaction terms for depression/anxiety/wellbeing and risky digital activity. Risky digital activity was mean‐centred. The models were conducted separately for depression, anxiety and wellbeing. In the models, the predictors were depression/anxiety/wellbeing, risky digital activity, and the interaction term of depression/anxiety/wellbeing and risky digital activity.

## 3 Results

### 3.1 RQ1: Perceived Impact of Online Experiences and Level of Concern

The majority of participants reported being concerned about the impact of their digital activity in general. However, for specific daily functioning, reports of frequent negative impacts were rare overall (see Table 1). Sleep was reported as most frequently affected, with a third of participants reporting sleeping less on *most days* or *every day*. Difficulties at school and missing meals were reported next most frequently. Social impacts concerning conflicts with family or friends and missing out on fun offline activities were rarely reported. In terms of mental health, 40% and 31%, respectively, reported that their mental health was positively or negatively affected by their digital activity *quite a lot* or *very much*. Further, 78% reported being worried to some degree about the impact of digital activity on their mental health.

**TABLE 1:** Percentage of individuals who thought that their digital activity had an impact on their mental health and functioning over the last 2 weeks (*n* = 383).

Daily functioning	Never	Just once, twice/a few times a week	Most days/at least every day
Slept less	16%	51%	33%
Had difficulties at school	48%	39%	13%
Missed meals	39%	47%	14%
Experienced conflicts with family	61%	32%	7%
Missed out on fun activities offline	62%	32%	6%
Experienced conflicts with friends	62%	33%	5%

Mental health	Not at all	Slightly/somewhat	Quite a lot/very much
Being online positively affected mental health	10%	50%	40%
Being online negatively affected mental health	17%	52%	31%
Worried how being online affected mental health	22%	50%	28%

### 3.2 RQ2: Attempts to Modify/Manage Online Experiences to Reduce the Negative Impact on Mental Health

Table 2 reports the proportion of individuals who undertook each action to manage their digital activity. Attempts to manage digital activity were very common, with 82% of participants engaging in *at least one approach at least once* in the last 2 weeks. Just under half of the participants reported engaging in an approach *most days or every day* (44%). The most commonly employed management actions involved shifting from negative to positive content. The least often reported were ‘look for friends online’ and ‘stop using the internet’.

**TABLE 2:** Percentage of individuals who undertook each specific action to manage their digital activity for mental health reasons, ranked from most to least common (*n* = 383).

Risk management actions	Never	Just once or twice/few times a week	Most days/at least every day
Engaged with positive content	18%	38%	44%
Looked for positive content	23%	43%	34%
Avoided negative people	28%	40%	32%
Avoided upsetting content	30%	41%	29%
Turned off notifications	35%	36%	29%
Viewed my experiences more positively	33%	42%	25%
Removed/blocked accounts	45%	41%	13%
Distracted myself from negativity online	51%	36%	13%
Took a break from social media	51%	38%	11%
Deleted messages/apps	55%	39%	6%
Reported a problem/account	62%	33%	5%
Sought advice/support	71%	24%	5%
Looked for friends online	73%	22%	5%
Stopped using internet	72%	24%	4%

### 3.3 RQ3: Factor Structure of Risk Management Actions

A three‐factor solution was optimal, accounting for 57.3% of the variance (see Table 3). Factor one was termed *Enhancing Positive Engagement,* which includes actions to increase positive content and avoid negative experiences and content. Factor two was termed *Coping Actions* and included items describing reporting/blocking accounts or problems. Factor three was termed *Reducing Engagement* and included stopping or reducing internet use. Cronbach's alphas for the three factors are 0.86, 0.71, and 0.70, respectively. Test‐retest reliability coefficients for the three factors are *r* = 0.58, *r* = 0.73, and *r* = 0.57, respectively.

**TABLE 3:** Item‐to‐factor loadings for the three types of risk management actions (*n* = 383).

Items	Factor		
	Enhancing Positive Engagement	Coping Actions	Reducing Engagement
Engaged with positive content	**0.847**	0.005	−0.088
Looked for positive content	**0.783**	−0.061	0.047
Viewed my experiences more positively	**0.703**	0.145	−0.086
Avoided negative people	**0.625**	0.234	0.007
Avoided upsetting content	**0.602**	−0.135	0.262
Reported a problem/account	−0.037	**0.685**	−0.042
Looked for friends online	−0.005	**0.548**	−0.012
Sought advice/support	0.033	**0.474**	0.094
Removed/blocked accounts	0.211	**0.431**	0.127
Distract myself from negativity online	0.308	**0.399**	0.096
Took a break from social media	0.081	−0.077	**0.772**
Stopped using internet	−0.090	0.117	**0.685**
Deleted messages/apps	0.054	0.313	**0.391**
Turned off notifications	0.282	0.020	0.292

*Note:* Loadings > 0.40 are shown in bold. Extraction Method = Principal Axis Factoring. Rotation Method = Oblimin with Kaiser Normalisation.

**FIGURE 1:**
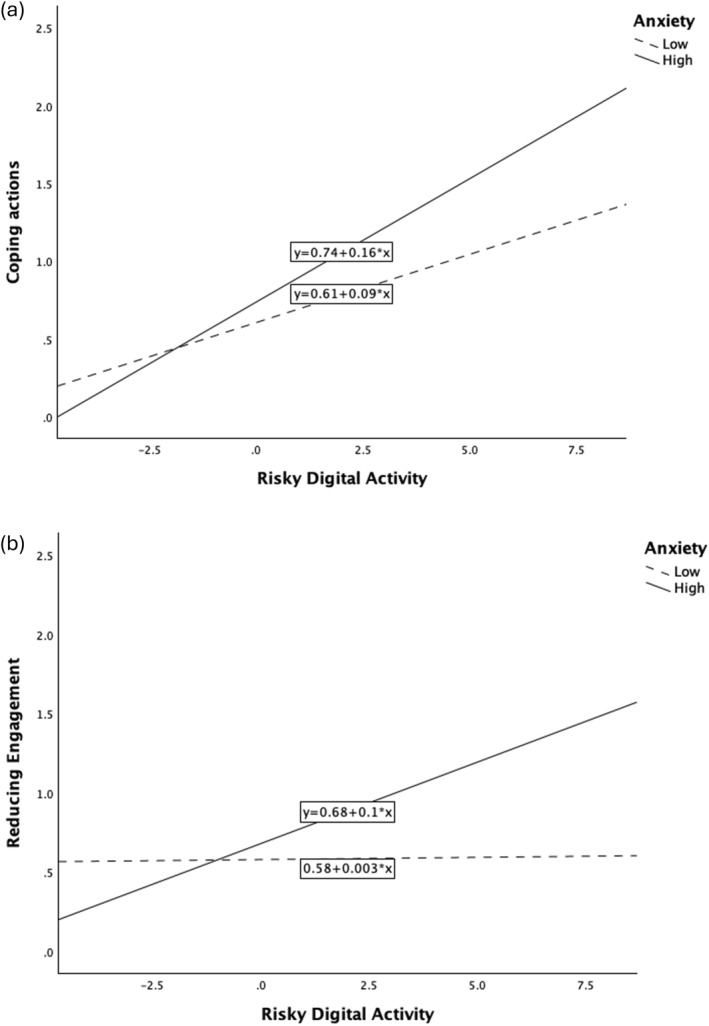
Interaction plots of the association between risky digital activity with Coping Actions (a) and Reducing Engagement (b) as the level of anxiety symptoms increases.

Across the three risk management actions, *Enhancing Positive Engagement* was most frequently adopted (mean = 1.72, SD = 1.08; adopted at least once by 82% of participants in the past 2 weeks), followed by *Coping Actions* (mean = 0.69, SD = 0.69; 55% participants). *Reducing Engagement* was the least adopted (mean = 0.66; SD = 0.76; 49% participants). Please see Supporting Information S1: Figure 1 for the mean scores for each risk management action factor. One‐way ANOVA suggested a significant effect (F(2, 1141) = 187.79, *p* < 0.001), with post hoc comparisons using the Bonferroni test showing that the mean of *Enhancing Positive Engagement* was significantly higher than the other two factors (both *p* < 0.001). There was no significant difference between *Coping Actions* and *Reducing Engagement.*

### 3.4 RQ4: Links Between Perceived Impact and Attempts to Modify/Manage Their Online Experiences

We aggregated the scores of the functional impact items based on the results of an EFA that suggested a single factor: functional impact (see Supporting Information S1: Table 1). Table 4 presents the correlations between the score on this factor and the factor scores for the three risk management actions. All correlations were significant across the small to moderate range. Correlations between the mental health impact items are presented in Supporting Information S1: Table 2. The associations for ‘*Negatively affected*’ and ‘*Worried about the impact*’ ratings were similar, with strongest associations for these being with *Enhancing Positive Engagement* (*r* = 0.29 and 0.33) and *Coping Actions* (*r* = 0.33 and 0.29). In contrast, perceived functional impact was most strongly correlated with *Coping Actions* (*r* = 0.37) and *Reducing Engagement* (*r* = 0.25)*. ‘Positively affected’* was most strongly associated with *Enhancing Positive Engagement* (*r* = 0.27).

**TABLE 4:** Bivariate correlations between the perceived impact of digital activity and risk management actions.

*n* = 382	Functional impact	Impact on mental health		
		Positive	Negative	Worried
Risk management actions				
Enhancing Positive Engagement	0.13*	0.27**	0.29**	0.33**
Coping Actions	0.37**	0.12*	0.33**	0.29**
Reducing Engagement	0.25**	0.14**	0.19**	0.23**

^**^ *p* < 0.01.

^*^ *p* < 0.05.

### 3.5 RQ5: Links Between Perceived Impact, Risk Management Actions, Mental Health, and Risky Digital Exposure

Perceived functional impact, ‘*Negatively affected*’ and ‘*Worried about the impact*’ were similarly correlated to a moderate degree with depression and anxiety (Table 5). *Coping Actions* and *Reducing Engagement* were positively correlated with depressive (*r* = 0.22 and 0.14) and anxiety (*r* = 0.33 and 0.18) symptoms. *Enhancing Positive Engagement* was correlated with anxiety and wellbeing (*r* = 0.19 and 0.26). Exposure to risky digital activity was correlated with higher perceived functional impact (*r* = 0.63), ‘*Negatively affected*’ (*r* = 0.50), ‘*Worried about the impact*’ (*r* = 0.41), and adopting all three risk management actions, most strongly *Coping Actions* (*r* = 0.43).

**TABLE 5:** Bivariate correlations between the perceived impact of digital activity, risk management actions, with mental health.

*n* = 378	Depression	Anxiety	Wellbeing	Risky digital activity
Functional impact	**0.55** **	**0.48** **	**−0.28** **	**0.63** **
Impact on mental health				
Positive	−0.03	0.01	**0.25** **	0.07
Negative	**0.44** **	**0.35** **	**−0.20** **	**0.50** **
Worried	**0.33** **	**0.31** **	−0.05	**0.41** **
Risk management actions				
Enhancing Positive Engagement	0.07	**0.19** **	**0.26** **	**0.14** **
Coping Actions	**0.22** **	**0.33** **	0.05	**0.43** **
Reducing Engagement	**0.14** **	**0.18** **	**0.16** **	**0.19** **

*Note:* Significant coefficients are in bold.

^**^ *p* < 0.01.

*p < 0.05.

Tables 6 and 7 report the results of the multiple regressions. For depressive symptoms, main effect models showed that risky digital activity, but not depressive symptoms, were associated with increased *Enhancing Positive Engagement* (*β* = 0.14, *p* = 0.026), *Coping Actions* (*β* = 0.44, *p* < 0.001), and *Reducing Engagement* (*β* = 0.16, *p* = 0.009; Table 6). There was no interaction effect between depression and risky digital activity on any of the three risk management actions.

**TABLE 6:** Associations between risky digital activity, depressive symptoms and risk management actions.

*n* = 376	β	*p*‐value
Main effects		
Enhancing Positive Engagement		
Depressive symptoms	−0.01	0.934
Risky digital activity	**0.14** *	0.026
Coping actions		
Depressive symptoms	−0.03	0.634
Risky digital activity	**0.44** **	< 0.001
Reducing engagement		
Depressive symptoms	0.05	0.437
Risky digital activity	**0.16** **	0.009
Interaction effects		
Enhancing Positive Engagement		
Depressive symptoms	0.01	0.985
Risky digital activity	**0.17** **	0.009
Depression × risky digital activity	−0.10	0.079
Coping actions		
Depressive symptoms	−0.03	0.606
Risky digital activity	**0.42** **	< 0.001
Depression × risky digital activity	0.04	0.491
Reducing engagement		
Depressive symptoms	0.05	0.471
Risky digital activity	**0.14** *	0.028
Depression × risky digital activity	0.05	0.340

*Note:* Significant coefficients are in bold.

Abbreviation: β, standardised coefficient beta.

^**^ *p* < 0.01.

^*^ *p* < 0.05.

**TABLE 7:** Associations between risky digital activity, anxiety symptoms and risk management actions.

*n* = 376	β	*p*‐value
Main effects		
Enhancing Positive Engagement		
Anxiety symptoms	**0.16** **	0.007
Risky digital activity	0.05	0.429
Coping actions		
Anxiety symptoms	**0.15** **	0.007
Risky digital activity	**0.34** **	< 0.001
Reducing engagement		
Anxiety symptoms	0.11	0.061
Risky digital activity	**0.13** *	0.031
Interaction effects		
Enhancing Positive Engagement		
Anxiety symptoms	**0.17** **	0.007
Risky digital activity	0.05	0.385
Anxiety × risky digital activity	−0.02	0.684
Coping actions		
Anxiety symptoms	**0.14** *	0.015
Risky digital activity	**0.31** **	< 0.001
Anxiety × risky digital activity	**0.12** *	0.017
Reducing engagement		
Anxiety symptoms	0.09	0.123
Risky digital activity	0.08	0.182
Anxiety × risky digital activity	**0.16** **	0.004

*Note:* Significant coefficients are in bold.

Abbreviation: β, standardised coefficient beta.

^**^ *p* < 0.01.

^*^ *p* < 0.05.

The results were different for anxiety symptoms. Main effect models for anxiety showed that higher anxiety was significantly associated with more *Enhancing Positive Engagement* (β = 0.16, *p* = 0.007) and *Coping Actions* (β = 0.15, *p* = 0.007) but not with *Reducing Engagement* (Table 7). There was a significant interaction between anxiety and risky digital activity on *Coping Actions* and *Reducing Engagement.* This suggested that individuals with higher anxiety who engaged in risky digital activity were more likely to engage in *Coping Actions* (*p* = 0.017 for interaction) and *Reducing Engagement* (*p* = 0.004 for interaction). Simple slopes for *Coping Actions* and *Reducing Engagement* are shown in Figure 1a and b, respectively. The level of anxiety symptoms was categorised as lower (below the median) and higher (above the median).

### 3.6 RQ6: Comparing the Effects on Mental Ill‐Health and Wellbeing

**FIGURE 2:**
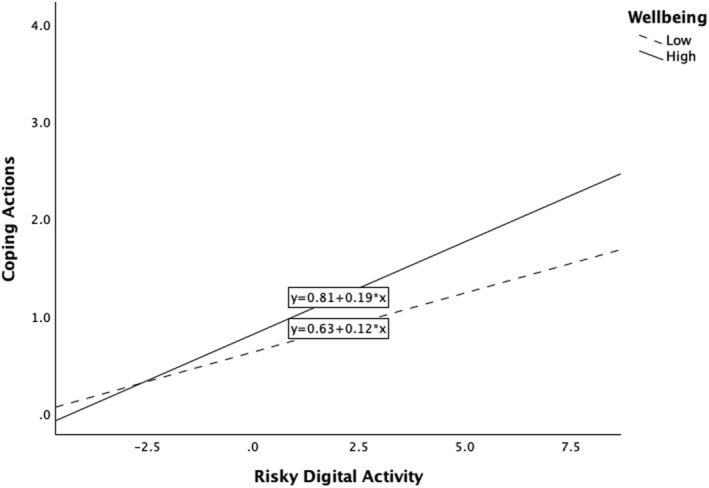
Interaction plot of the association between risky digital activity with Coping Actions as the level of wellbeing increases.

Main effect models for wellbeing (Table 8) showed that higher wellbeing was significantly associated with more *Enhancing Positive Engagement* (β = 0.30, *p* < 0.001), *Coping Actions* (β = 0.15, *p* = 0.002), and *Reducing Engagement* (β = 0.22, *p* < 0.001). There was a significant interaction between wellbeing and risky digital activity on *Coping Actions.* This suggested that individuals with higher wellbeing who engaged in risky digital activity were more likely to engage in *Coping Actions* (*p* = 0.007 for interaction). Simple slopes for *Coping Actions* are shown in Figure 2. The level of wellbeing was categorised as lower (below the median) and higher (above the median).

**TABLE 8:** Associations between risky digital activity, wellbeing and risk management actions.

*n* = 372	β	*p*‐value
Main effects		
Enhancing Positive Engagement		
Wellbeing	**0.30** **	< 0.001
Risky digital activity	**0.19** **	< 0.001
Coping actions		
Wellbeing	**0.15** **	0.002
Risky digital activity	**0.45** **	< 0.001
Reducing engagement		
Wellbeing	**0.22** **	< 0.001
Risky digital activity	**0.24** **	< 0.001
Interaction effects		
Enhancing Positive Engagement		
Wellbeing	**0.29** **	< 0.001
Risky digital activity	**0.19** **	< 0.001
Wellbeing × risky digital activity	0.07	0.153
Coping actions		
Wellbeing	**0.14** **	0.005
Risky digital activity	**0.45** **	< 0.001
Wellbeing × risky digital activity	**0.13** **	0.007
Reducing engagement		
Wellbeing	**0.21** **	< 0.001
Risky digital activity	**0.24** **	< 0.001
Wellbeing × risky digital activity	0.09	0.080

*Note:* Significant coefficients are in bold.

Abbreviation: β, standardised coefficient beta.

^**^ *p* < 0.01.

*p < 0.05.

## 4 Discussion

The goal of the present study was to advance understanding of young people's awareness of and agency in managing their digital engagement to minimise its negative impacts and maximise its positive impacts on their mental health and wellbeing, through the development, validation, and application of a novel measure: the DIAS. Based on the DIAS, most participants expressed some degree of recognition and worry about the negative impacts of their recent online experiences, with around 30% reporting significant concerns (MacKenzie et al. 2022; Popat and Tarrant 2023). Our findings suggest that young people reported both positive (90%) and negative (83%) impacts of digital activity at similarly high rates. Rather than indicating that digital activity is viewed as predominantly positive or negative, this pattern highlights a nuanced and ambivalent appraisal, in that young people hold mixed and sometimes contradictory views about their digital lives, with both positive and negative experiences co‐existing. These findings reflect the complexity of young people's digital experiences, contrasting with a more polarised public discourse that often emphasises predominantly negative effects. On a positive note, this awareness is associated with agency to manage these impacts. Our results showed that a majority of young people actively managed their online activities: a potential intervention target that is arguably neglected in current efforts to address negative impacts on digital engagement, which tend to focus on restricting access and reducing time online.

Interestingly, the degree of perceived positive impact was neither correlated with an absence of recent risky digital engagement (as measured by the DAFI), nor linked to lower depressive and anxiety symptoms. This suggested that the positive outlook was not based on recent digital activity or mental health experiences. Moreover, it was associated with more engagement in *all three* types of risk management action. This seems counterintuitive: why would a young person attempt to reduce the mental health risks associated with digital activity when they perceive a positive impact on their mental health? One possible explanation refers to the complexity of young people's digital activities and their concurrent but sometimes diverging effects—while some aspects of their digital activity might have induced positive impacts (e.g., social engagement and leisure activities), others might have induced more negative effects (e.g., risky content and interactions) and require risk management actions.

In terms of the impact of digital activity on daily functioning, negative impacts were reported by fewer young people. The exception was sleep, with more than 30% expressing a relatively high degree of impact—being affected most days or every day. This is in line with a growing body of literature on the detrimental effect of digital activity on the quantity and quality of sleep in young people (LeBourgeois et al. 2017; Lemola et al. 2015; Pagano et al. 2023) and how this may mediate downstream effects on academic achievement and mental health (Husarova et al. 2018; Mao et al. 2022). In this regard, it is interesting that the next most frequently perceived functional impact was ‘difficulties at school’—which may, in turn, be linked to lack of sleep (Pérez‐Chada et al. 2023). Although the levels of reported impact are much lower than for sleep, the finding that around 60% had missed meals is another source of concern, given the importance of a healthy diet to both mental and physical health (Mathers et al. 2009). Finally, although negative impacts on social relationships with friends and parents were relatively rare, they may have a detrimental downstream effect on mental health when they do occur (Kowert et al. 2014).

As well as recognising the risks of recent digital activity, most participants reported that they had, over the previous 2 weeks, engaged in some risk management actions to actively address such concerns. This is important, as it highlights the importance of youth agency in mitigating digital risks. Factor analysis suggested three dimensions of risk management actions, the first two of which related to actions taken to change the online experience rather than reducing time spent online. The first, and most frequently adopted, set of actions (*Enhancing Positive Engagement*) involved shifting the balance of digital activity from negative to positive content/activities and/or adopting a more positive mindset more generally. The second set of actions (*Coping Actions*) involved various strategies for mitigating risk, including seeking advice and support and attempts to manage harmful content (block accounts/report a problem). The final set of actions involved reducing, but not completely cutting out, time spent online (*Reducing Engagement*)—commonly referred to as ‘digital detox’ (Radtke et al. 2022). However, the correlations with mental health and wellbeing were only moderate in strength, suggesting that many concerned young people did not attempt to reduce their overall digital activity. It would be valuable in future longitudinal research to examine whether young people engage in a stepped approach to risk management—for example, starting with enhancing positive actions and then moving to more targeted risk management (e.g., coping), or use several approaches at the same time.

The study also found that the more worried a young person is about the impact of their digital activity on their mental health, the more likely they are to engage in risk management actions. The associations were similar for the three risk management action domains, suggesting that young people who are worried may adopt a range of strategies. Similarly, higher recent levels of risky digital activity, as measured by the DAFI (risky content, risky interaction and social comparisons), were associated with greater worry about the negative impacts and more actions to mitigate the risk. These associations provide an important validation of the DIAS, supporting its construct and predictive validity.

Finally, wellbeing was significantly associated with all three risk management actions, even more strongly than depression and anxiety were. This suggested a strong link between digital risk management and wellbeing, although we do not know to what extent adopting these management actions promotes a better wellbeing versus wellbeing facilitates engaging in risk management. Existing research has explored the differential causal pathways for wellbeing in contrast with depression and anxiety, highlighting them as correlated but distinct factors (Keyes 2005; Kinderman et al. 2015). However, longitudinal research is needed to clarify the directionality between digital risk management, mental health, and wellbeing.

Future longitudinal research will also be necessary to examine whether the risk management actions are successful. In the present study, depression and especially anxiety were correlated with concerns about the mental health risks of digital activity and the likelihood of engaging in risk management actions. Anxiety also appeared to exacerbate the impacts of risky digital exposure as a driver of awareness and attempts to reduce those risks. However, it is likely that recognition of digital risks to mental health, attempts to mitigate that risk by engaging in risk management actions, and levels of depression and anxiety are related to each other in a dynamic and multifaceted way—with increased mental health difficulties leading to risk mitigation strategies and then to subsequent improvements in mental health. It would also be interesting to link DIAS data to objective measures of digital activity and sleep to compare the objective and subjective effects.

This is the first exploration of relationships between an individual's recognition of the negative impacts of digital activity on their mental health and their agentic decisions to undertake digital risk management actions to mitigate these negative impacts. However, the study has several limitations. First, as a cross‐sectional study, the direction of effects cannot be addressed. Second, the results were based on a convenience sample (albeit one showing a high level of diversity), therefore, the rates of worry related to digital risks and different risk management activities may not generalise to the wider youth population. Nevertheless, the correlations might be even stronger in the general population, given that our sample had slightly privileged socioeconomic status. Third, the study relied on self‐reports, increasing the risk of shared method variance, which can inflate correlations between variables. The reports of digital activities may also be influenced by social desirability by under‐reporting negative and risky activities. However, subjective experiences may be more predictive of later mental health problems than objective indices (Danese and Widom 2023). Future research would benefit from recruiting a larger and more representative youth sample and supplementing self‐report with proxy‐report by a parent/caregiver, friend, or sibling. Moreover, the DIAS is designed to capture the general impacts of online experiences, rather than effects and actions tied to specific digital activities. To explore the source of the perceived negative impact, we included a separate questionnaire, the Digital Activity and Feelings Inventory (DAFI), which captures distinct types of digital activities. Our findings highlight strong associations between risky digital exposure (e.g., risky interactions, social comparisons, inappropriate content) and perceived negative impacts. Building upon this, future research could examine connections between individual digital activities and their impacts, particularly the positive ones.

In summary, using data from the newly developed DIAS, this study provided important findings about the role of insight and agency in young people's attempts to manage the impact of online experiences on their mental health and daily functioning. The study also provided a validation of the new measure, supporting its reliability and a three‐factor structure. Our data provide a preliminary basis on which to develop new intervention approaches working with the grain of young people's existing strategies, which may help reduce negative mental health outcomes associated with certain forms of digital activity. It also motivates future longitudinal research to tease apart the directionality of effects and to understand the development and longer‐term effects of these risk management actions. Our findings and introduction of a novel tool is a vital step towards framing and evaluating interventions that can help improve adolescent mental health in the digital age.

## Author Contributions

**P. Tang:** conceptualisation, writing – original draft, writing – review and editing, data curation, formal analysis, project administration. **K. Kostyrka‐Allchorne:** conceptualisation, writing – original draft, writing – review and editing, funding acquisition, supervision. **J. Bourgaize:** conceptualisation, writing – review and editing, data curation, project administration. **A. Murray:** conceptualisation, writing – review and editing. **M. Stoilova:** conceptualisation, writing – review and editing. **M. E. Etherson:** conceptualisation, writing – review and editing. **E. Azeri:** writing – review and editing, data curation. **I. Abbas:** writing – review and editing, data curation, project administration. **A. Bridgwood:** writing – review and editing, data curation, project administration. **C. Hollis:** writing – review and editing, funding acquisition. **E. Townsend:** writing – review and editing, funding acquisition. **S. Livingstone:** conceptualisation, writing – original draft, writing – review and editing, funding acquisition, supervision. **E. J. S. Sonuga‐Barke:** conceptualisation, writing – original draft, writing – review and editing, funding acquisition, supervision.

## Funding

The authors (P.T., K.K.‐A., J.B., A.M., M.S., M.E., A.E., I.A., A.B., C.H., E.T., S.L. and E.S.‐B.) acknowledge the support of the UK Research and Innovation (UKRI) Digital Youth Programme award (Medical Research Council) as part of Digital Youth ‐ a large multi‐institutional programme of research https://digitalyouth.ac.uk (MR/W002450/1; chief investigators: C.H. and E.T.), which is part of the Adolescence, Mental Health and the Developing Mind programme. This study is in part funded by the National Institute for Health and Care Research (NIHR) Maudsley Biomedical Research Centre (BRC). The views expressed are those of the author(s) and not necessarily those of the NIHR or the Department of Health and Social Care.

## Conflicts of Interest

The authors declare no conflicts of interest.

## Supporting information


Supporting Information S1

## Data Availability

The data will be deposited in a public archive within 2 years after the end of the project (current project end date: 31 Mar 2026).
