# Supplementary material for: ‘Digital Insight and Agency Scale’ (DIAS): A Novel Tool to Illuminate Young People's Agency in Mitigating the Negative Impact of Digital Activities on Their Mental Health
Source: Int J Methods Psychiatr Res. 2026 Feb 9;35(1):e70053. doi: 10.1002/mpr.70053 (PMC12884440; doi:10.1002/mpr.70053)
Supplement: Supplementary file 1 — Supporting Information S1 [file MPR-35-e70053-s001.docx]

**Title:** ‘Digital Insight and Agency Scale’ (DIAS): a novel tool to illuminate young people’s agency in mitigating the negative impact of digital activities on their mental health.

# Supplementary materials


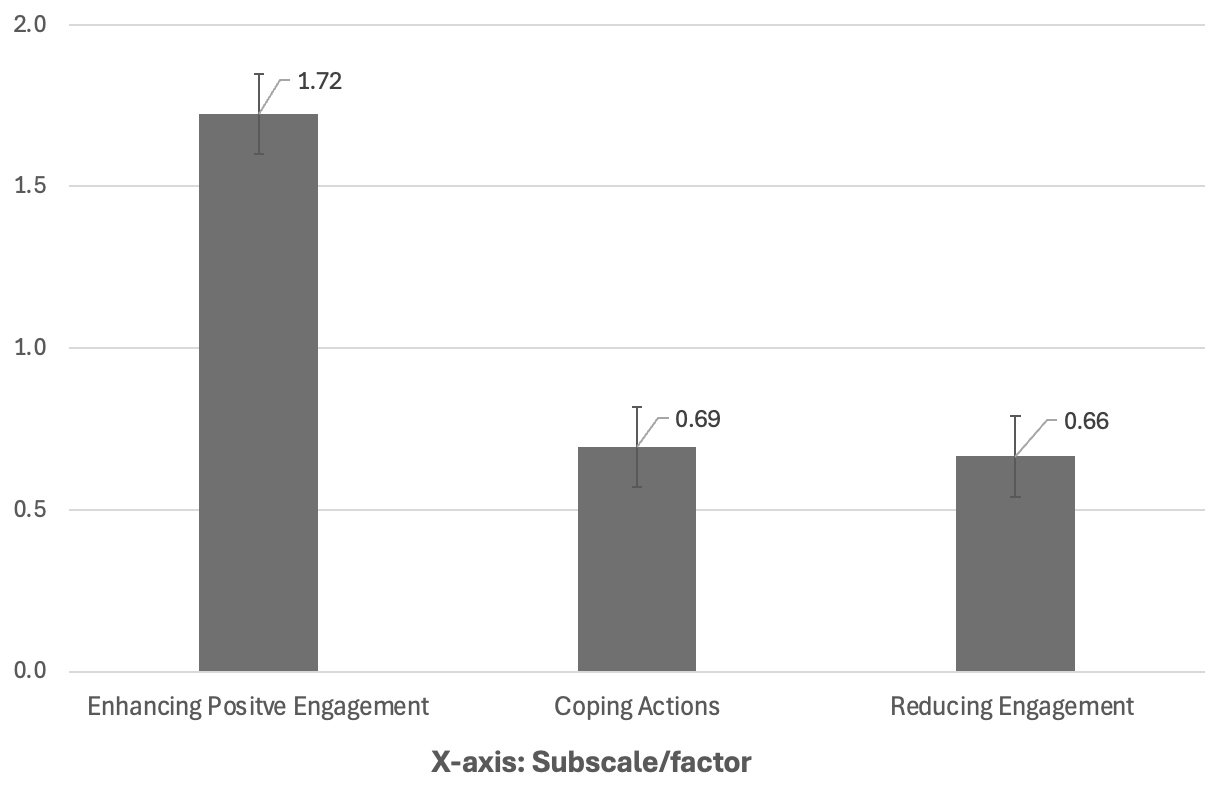


Supplementary Figure 1. The item means for the three risk management actions.

Supplementary Table 1. Item-to-factor loadings for the functional impact items (DIAS section one)

| **Items** | **Factor** |
| --- | --- |
|  | *Functional impact* |
| Missed meals | .556 |
| Slept less | .360 |
| Family conflict | .686 |
| Friend conflict | .649 |
| School problems | .683 |
| Missing out on fun activities offline | .670 |

Note. Extraction Method = Principal Axis Factoring. Rotation Method = Oblimin with Kaiser Normalization.

Supplementary Table 2.Correlations between mental health impact items (DIAS section one)

| **Items** | 1 | 2 |
| --- | --- | --- |
| 1. Being online positively affected my mental health’ |  |  |
| 1. Being online negatively affected my mental health’ | .140** |  |
| 1. I worried how being online affected my mental health | .116* | .684*** |

Note. *** *p* < .001, ** *p* < .01, * *p* < .05.
